# Herbal Medicine for Oligomenorrhea and Amenorrhea: A Systematic Review of Ancient and Conventional Medicine

**DOI:** 10.1155/2018/3052768

**Published:** 2018-03-18

**Authors:** Arezoo Moini Jazani, Kobra Hamdi, Mojgan Tansaz, Hossein Nazemiyeh, Homayoun Sadeghi Bazargani, Seyed Mohammad Bagher Fazljou, Ramin Nasimi Doost Azgomi

**Affiliations:** ^1^Department of Iranian Traditional Medicine, School of Traditional Medicine, Tabriz University of Medical Sciences, Tabriz, Iran; ^2^Women's Reproductive Health Research Center, Tabriz University of Medical Sciences, Tabriz, Iran; ^3^Department of Traditional Medicine, School of Traditional Medicine, Shahid Beheshti University of Medical Sciences, Tehran, Iran; ^4^Research Center for Pharmaceutical Nanotechnology, Faculty of Pharmacy, Tabriz University of Medical Sciences, Tabriz, Iran; ^5^Road Traffic Injury Research Center, Tabriz University of Medical Sciences, Tabriz, Iran

## Abstract

**Introduction:**

Menstrual bleeding cessation is one of the most frequent gynecologic disorders among women in reproductive age. The treatment is based on hormone therapy. Due to the increasing request for alternative medicine remedies in the field of women's diseases, in present study, it was tried to overview medicinal plants used to treat oligomenorrhea and amenorrhea according to the pharmaceutical textbooks of traditional Persian medicine (TPM) and review the evidence in the conventional medicine.

**Methods:**

This systematic review was designed and performed in 2017 in order to gather information regarding herbal medications of oligomenorrhea and amenorrhea in TPM and conventional medicine. This study had several steps as searching Iranian traditional medicine literature and extracting the emmenagogue plants, classifying the plants, searching the electronic databases, and finding evidences. To search traditional Persian medicine references, Noor digital library was used, which includes several ancient traditional medical references. The classification of plants was done based on the repetition and potency of the plants in the ancient literatures. The required data was gathered using databases such as PubMed, Scopus, Google Scholar, Cochrane Library, Science Direct, and web of knowledge.

**Results:**

In present study of all 198 emmenagogue medicinal plants found in TPM, 87 cases were specified to be more effective in treating oligomenorrhea and amenorrhea. In second part of present study, where a search of conventional medicine was performed, 12 studies were found, which had 8 plants investigated:* Vitex agnus-castus, Trigonella foenum-graecum, Foeniculum vulgare, Cinnamomum verum, Paeonia lactiflora, Sesamum indicum, Mentha longifolia,* and* Urtica dioica. Conclusion*. Traditional Persian medicine has proposed many different medicinal plants for treatment of oligomenorrhea and amenorrhea. Although just few plants have been proven to be effective for treatment of menstrual irregularities, the results and the classification in present study can be used as an outline for future studies and treatment.

## 1. Introduction

Oligomenorrhea and amenorrhea are one of the most common gynecologic complaints among women in reproductive age. Prevalence of oligomenorrhea and secondary amenorrhea has been reported to be 10–15 percent and 3-4 percent, respectively [1, 2]. Regardless of the diagnosis, oligomenorrhea and amenorrhea are changes in regular menstrual cycles which include long menstrual cycles and absence of menstruation, respectively [3]. Hormonal therapy based on estrogen and progesterone compounds is the mainstay of the treatment for these conditions [4].

Nowadays there has been an increasing trend in usage of complementary and alternative medicine (CAM) [5]. Traditional Persian medicine (TPM), as a holistic system of medicine and based on temperament, is one of the wealthiest branches of the CAM and has been used in Iran since thousands years ago [6]. Temperament is made of action and reaction of four pivotal elements (fire, air, water, and soil) and creates different characteristics in living things [7]. In TPM, temperament has been classified in different types: hot, cold, wet, and dry [8]. Any disturbances in normal temperament of organs (as said mal-temperaments) can cause diseases. Treatment is based on modifying the temperament [9]. Amenorrhea, oligomenorrhea, and hypomenorrhea are defined as “*Ehtebas Tams*” in TPM. From TPM viewpoint, anatomical and functional disorders (mal-temperaments) in the uterus and ovaries and other organs aside from the genital system are the main causes of oligomenorrhea and amenorrhea [10]. The treatment of oligomenorrhea and amenorrhea includes lifestyle modification (especially nutritional habits and diet, physical activities, and sleep), medication, and nonmedical methods like wet and dry cupping and surgery [11, 12]. Herbal medications are one of the key tenets for treatment and can modify mal-temperaments of the organs [11, 12].

The aim of our study was to overview medicinal plants used to treat oligomenorrhea and amenorrhea according to the medical and pharmaceutical textbooks of TPM and review the evidence in the conventional medicine in order to recommend more efficient treatment guidelines for the research priorities in the future and also help to treat the patients by new pharmaceutical formulations.

## 2. Methods

This systematic review was designed and performed in 2017 in order to gather information regarding herbal medications of oligomenorrhea and amenorrhea in TPM and conventional medicine. This study had five steps: (1) searching Iranian traditional medicine literature and extracting the emmenagogue plants; (2) classification of the plants; (3) searching and extracting the scientific name of the plants; (4) searching the electronic databases and finding evidences; (5) ranking the data found in the studies. To search TPM references, Noor digital library was used, which includes several ancient traditional medical references (Table 1). This valuable database contains more than thousand Islamic and TPM books. The main keywords were “*Moder Heiz or Tams,*” “*Ehtebas Heiz or Tams,*” “*Tams,*” “*Heiz,*” and any words in Persian or Arabic that meant causing menstrual bleeding. In this part, the emmenagogue plants were identified and selected. The traditional name, temperament, and the exact phrases about its function in reproductive system and occurring menstruation were extracted.

The medicinal plants listed in the past step were classified in second step. The classification of plants was done based on the repetition and potency of the plants in the ancient literatures. A repetitious plant was defined as a plant which was repeated at least in 5 pharmaceutical textbooks of traditional Persian medicine (Table 1) [11, 13–16, 18, 19, 27]. In addition, the potent plants were isolated from the preliminary list. Potent, experienced, specific, unique plants were defined as medicinal herbs which were powerful or special or experienced in menstrual induction according to traditional Persian medicine scientists' concept. The used part and application method of the plants were also extracted.

In third step, the scientific name of frequent and potent plants was quested and extracted from some botanical reference books, databases, and articles such as “Popular Medicinal Plants of Iran” [20], “Matching the Old Medicinal Plant Names with Scientific Terminology” [21], “Dictionary of Iranian Plant Names” [22], “Dictionary of Medicinal Plants” [23], and “The Plant List” [40].

In fourth step, to investigate relevant information in conventional medicine required data was gathered by using databases such as PubMed, Scopus, Google Scholar, Cochrane Library, Science Direct, and web of knowledge. Time period between 2000 and 2017 was selected. Also, to increase scope of study, manual search in some of the valid journal databases was performed. The search terms were the scientific/common name of each plant in the whole text AND “oligomenorrhea” OR “amenorrhea” OR “polycystic ovary” OR “PCOs” OR “ovarian” OR “mense” OR “menstruation” OR “menstrual” OR “emmenagogue” in title/abstract. As far as polycystic ovary syndrome is known to be one of the main causes of oligomenorrhea and secondary amenorrhea, articles regarding use of herbal medicine in polycystic ovary syndrome were also included in the study to enrich the articles collection. In the fifth step, the ranking will be based on the data extracted from steps two to four.

**Figure 1:**
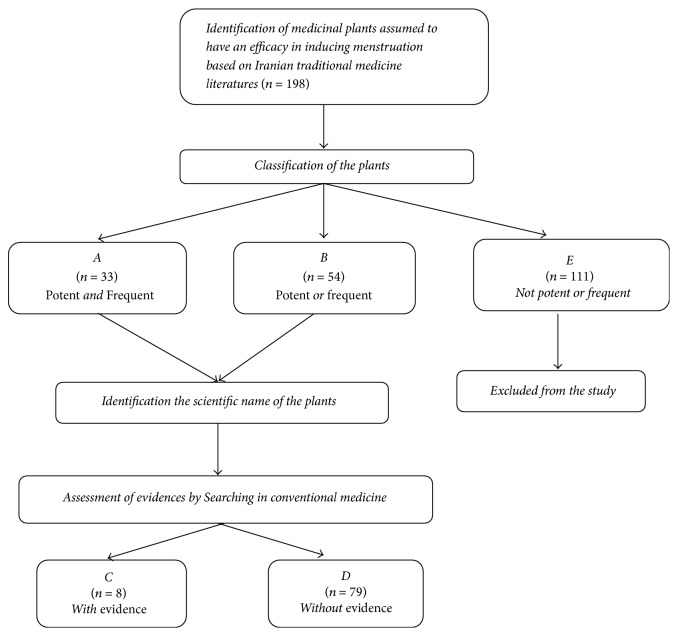
Flowchart regarding classification of medicinal plants used for treatment of oligomenorrhea and amenorrhea.

One reviewer extracted the data from the included studies while a second author checked the results. Any disagreements were resolved by discussion of reviewers. Data for the primary objective of the review was collected from the full text of each publication and included the trial name, year of publication, type of study, sample size, results, and other details. Flowchart regarding classification of the medicinal plants is shown in Figure 1.

Statistical analysis was performed by SPSS software package version 16.0 for windows (SPSS Inc., Chicago, USA).

## 3. Results

**Figure 2:**
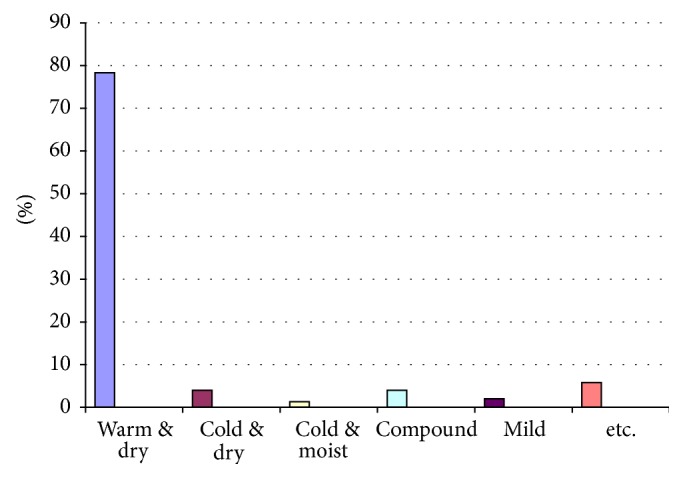
Proportion of temperament of medicinal plants found in present study.

In present study of all 198 herbs found in TPM [20–22, 24], 87 medicinal plants were found to be potent and frequent in treating oligomenorrhea and amenorrhea (Table 4); families Apiaceae (15.11%) and Lamiaceae (12.79%) were the most prevalent ones. Proportion of temperaments of these plants are shown in Figure 2. Based on the search of TPM, 70 medicinal plants were mentioned frequently to be effective in treatment of oligomenorrhea and amenorrhea and 50 medicinal plants were known as potent medicine for oligomenorrhea and amenorrhea treatment. Of all these plants, 33 were both frequently mentioned and potent (Tables 3 and 4).

**Figure 3:**
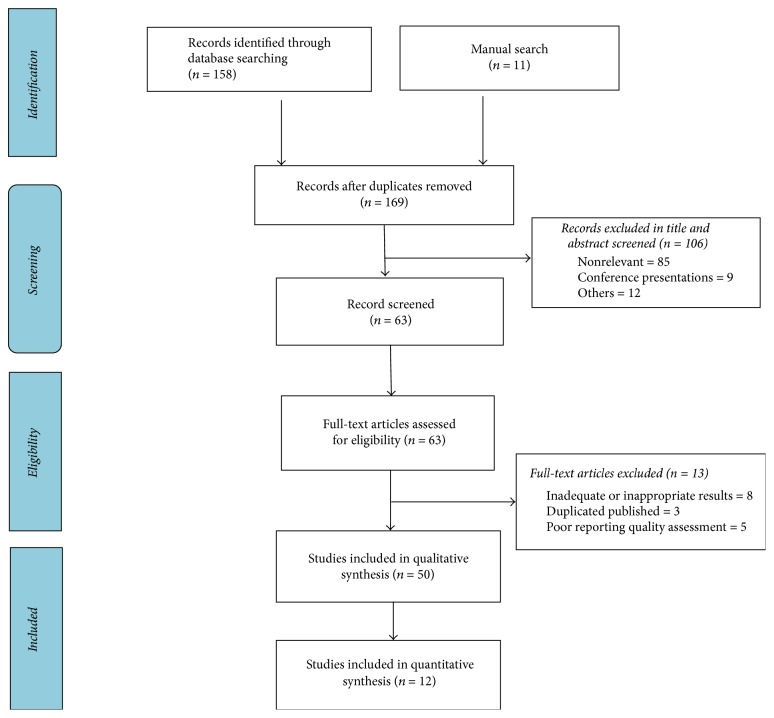
Flowchart of the systematic review of conventional medicine references to assemble studies regarding herbal medicines used for treatment of oligomenorrhea and amenorrhea.

In second part of present study, where a search of conventional medicine was performed, 12 studies were found (Table 5) which had 8 plants investigated:* Vitex agnus-castus, Trigonella foenum-graecum, Foeniculum vulgare, Cinnamomum verum, Paeonia lactiflora, Sesamum indicum, Mentha longifolia, *and* Urtica dioica*. Details of included studies are shown in Table 5.* Nigella sativa, Thymus serpyllum, Matricaria chamomilla, Pimpinella anisum, Marrubium vulgare, Ziziphora clinopodioides, Origanum majorana, *and* Allium cepa* are of the medicinal plants found to be effective in treatment of polycystic ovary syndrome; no therapeutic effect for oligomenorrhea and amenorrhea was mentioned [41–48]. The flowchart of the systematic review is shown in Figure 3.

### 3.1. *Vitex agnus-castus*

*Vitex agnus-castus* or* chasetree* belongs to family Lamiaceae, which has been used as a common medicine for treatment of menstrual irregularities and infertility since 2000 years ago [38, 39]. The effects of low dose estrogen and* Vitex agnus-castus* on the normalization of the menstrual cycle and the means of serum prolactin and free testosterone levels in the women with polycystic ovary syndrome were similar [39].

### 3.2. *Trigonella foenum-graecum*

*Trigonella foenum-graecum* is an annual plant belonging to family Fabaceae found in Mediterranean region and Iran. It has been traditionally used for gastrointestinal, pulmonary, and gynecologic diseases [19]. Based on the studies, this medicinal plant has been found effective in regulating menstrual cycles, improving fertility, and reversing features of polycystic ovary syndrome by decreasing LH to FSH ratio and reversing ultrasonographic features of it [35, 36].

### 3.3. *Cinnamomum verum*

*Cinnamon* is an evergreen plant from family Lauraceae with aromatic characteristics basically used as a condiment. In TPM, this plant is known as a potent medicine to increase libido and regulate menstruation and is effective in treating brain and pulmonary diseases [19]. Anti-inflammatory, antioxidative, antidiabetic, and lipid lowering features of* cinnamon* has been already proven [49].* Cinnamon* is known to increase serum progesterone level in luteal phase, so facilitating menstrual cycle regulation [28].

### 3.4. *Paeonia lactiflora Pall*

*Paeonia lactiflora Pall* is a medicinal plant used in Japan, Korea, China, and Iran since 1200 years. It has been used to cure stomachache, headache, hepatitis, dysmenorrhea, and menstrual cycle irregularities [19]. It has been already reported that a mixture of* Paeonia lactiflora *and* Glycyrrhiza glabra* extracts was effective in normalizing menstrual cycles and hormonal balance among women with polycystic ovary syndrome [32].

### 3.5. *Foeniculum vulgare*

*Foeniculum vulgare* or Fennel is a flowering plant from family Apiaceae which has been used as a medicinal plant in TPM due to its known antioxidative, anti-inflammatory, estrogenic, diuretic, emmenagogue, antithrombotic, and antihypertensive features [26]. This plant has been found to be effective in inducing menstruation after medroxyprogesterone acetate use in comparison to control group [29].

### 3.6. *Mentha longifolia*

*Mentha longifolia* is one of many members of family Lamiaceae which is used in pharmaceutical and industrial fields. This medicinal plant has been used to relieve gastrointestinal and gynecologic complaints for many years [19, 50]. It has been shown that using* Mentha longifolia* extracts induces menstruation and regulates menstrual cycles [30]. Also it has been indicated that this extract decreases FSH and induces menstruation among patients with primary ovarian failure [31].

### 3.7. *Sesamum indicum*

*Sesamum indicum* is a flowering plant in the genus* Sesamum* and family Pedaliaceae. In TPM, this medicinal plant has been used to increase libido, induce menstrual bleeding, and treat renal and pulmonary diseases [34]. Also,* sesame* has been found to have antihypertensive, antioxidant, and cholesterol lowering characteristics [33, 34].* Sesame* is known to induce menstruation without prominent side effects among women with severe oligomenorrhea [33, 34].

### 3.8. *Urtica dioica*

*Urtica dioica* is a herbaceous perennial flowering plant in the family Urticaceae, which has been used as a diuretic and to treat rheumatic diseases and arthritis [37]. Ancient Iranian physicians used* Urtica dioica* to increase libido, induce menstruation, and treat renal and pulmonary diseases [19]; also this plant has been found to have antioxidant, anti-inflammatory, antidiabetic, and antiandrogenic features; although based on a study* Urtica dioica* extract improved the clinical and paraclinical symptoms of hyperandrogenism in women, improvement of menstrual irregularities was lower in the intervention group compared to the control group [37].

## 4. Discussion

It deems that diseases of female reproductive system are one of the greatest challenges for modern medicine. Menstrual irregularities as one of the most frequent gynecologic complaints can affect the several aspects of women's health including their physical, mental, and social health [51, 52]. Oligomenorrhea and its different etiologies, especially PCOs, can lead to various complications such as infertility, pregnancy complications, cardiovascular disease, metabolic diseases like diabetes, hypertension, and fatty liver, and psychological disorders such as anxiety and depression and reduce quality of life in women [52–54].

Nowadays, due to some complications of hormonal therapy, many women have considered using alternative and complementary medicine [55, 56]. TPM is known as one of the main branches of alternative and complementary medicine, which tries to treat illnesses with change in lifestyle and using medicinal plants [57].

In present study, emmenagogue plants used to treat oligomenorrhea and amenorrhea were systemically searched. Based on current study, 33 plants were proven to be more effective (due to their potency and frequency in ancient literatures) in treatment of oligomenorrhea and amenorrhea in TPM (group A in Figure 1 and Table 3) as* Prangos ferulacea *L.*, Ferula persica *Willd*., Mentha longifolia, Artemisia absinthium, Thymus vulgaris, Phaseolus vulgaris, Ziziphora clinopodioides *Lam., and so on according to Table 3. The most prevalent temperaments of the plants were warm and dry. Eight plants were found to be effective in conventional medicine references (group C in Figure 1 and Table 3):* Cinnamomum verum, Foeniculum vulgare, Mentha longifolia, Paeonia lactiflora, Sesamum indicum *L*., Trigonella foenum-graecum, Urtica dioica, *and* Vitex agnus-castus*. Of all these plants, 5 plants belonged to both groups (groups A and C):* Foeniculum vulgare, Mentha longifolia, Paeonia lactiflora, Sesamum indicum *L*., *and* Vitex agnus-castus.* The plants of group C did not have any serious side effects in the dosage and duration of use according to reviewed articles in present study. Phytochemical studies have shown that flavonoids (quercetin, apigenin, and vitexin), phenols (anethole and thymol), phytosterols (stigmasterol and sitosterol), lignans, and terpenoids are of the main components in these medicinal plants which are responsible for their medicinal activities [58]. Although the exact mechanisms of these plants on oligomenorrhea are not fully understood, the antioxidant and anti-inflammatory properties of these herbs are likely to be one of the main mechanisms of their function. The anti-inflammatory and antioxidative features of the plants have been proposed to play the key role in regulating sex hormones, increasing insulin sensitivity, and modifying lipid profile [28, 50, 59, 60]. It has been reported that some of these plants contain phytoestrogenic components which lowers LH via a negative feedback process and decreases testosterone [61, 62]. Milewicz et al. showed that consumption of* Vitex agnus-castus* extract over a period of 3 months can reduce the prolactin release in latent hyperprolactinemia without significant side effect [63]. It has been proposed that some phytoestrogenic components in these plants with similar actions to natural sex hormones enhance follicle maturation, reduce coagulation factors, relax uterine muscles, and facilitate uterine recovery [29, 34, 64, 65].

Due to the paucity of studies on medicinal plants in the treatment of oligomenorrhea, the ranking of the plants in this study can be used to conduct further studies with a higher priority and also to treat the patients. According to the rankings (Table 2), the first group of the plants which was potent and frequent and have herbal studies evidences can be used as an outline for future studies and treatment of patients. Design of in vitro, animal, and even clinical studies with more proper quality and larger sample size is recommended to reveal exact mechanisms of these plants and manufacture new pharmaceutical products with minimal side effects. Group 2 plants, which did not have enough evidence, are at the second rank which has the priority to be studied in clinical and preclinical settings in order to evaluate the efficacy, mechanisms of activities, safety, and any probable side effects. Groups 3 and 4 because of lack of evidence are at the next research priorities.

Polycystic ovary syndrome is a set of symptoms in women which includes irregular or no menstrual periods, excess body and facial hair, acne, and infertility [66]. In present study*, Nigella sativa, Thymus vulgaris, Matricaria chamomilla, Pimpinella anisum, Marrubium vulgare, Ziziphora clinopodioides, Origanum majorana, *and* Allium cepa* were found to be effective in treatment of polycystic ovary syndrome, but there were not any reports about the treatment of oligomenorrhea and amenorrhea. Due to the effects of the mentioned herbs in the treatment of clinical and paraclinical symptoms of PCOs, these plants also may be proposed as a potent treatment for oligomenorrhea and amenorrhea.

One of the main limitations of present study was the lack of the resources from the other alternative and complementary medicine references, such as those used in China; inclusion of information from those references would have enriched the present study in a way that a more concise conclusion could have been made.

## 5. Conclusion

In present study, it was tried to assemble the available evidence about effect of medicinal plants on treating oligomenorrhea and amenorrhea in Persian and conventional medicine references. At last 5 plants were found to be strongly suggested in TPM and also proven to be effective in conventional medicine references:* Foeniculum vulgare, Mentha longifolia, Paeonia lactiflora, Sesamum indicum *L*., *and* Vitex agnus-castus*. This result can be utilized in clinical fields, by selecting more efficient, with less side effects, medicinal herbs. Although a lot of emphasis has been made about plants in alternative and complementary medicine, unfortunately there have not been enough studies in conventional medicine. Results of present study can be used as an outline for future studies about the plants found to be effective in conventional and complementary medicine.

## Figures and Tables

**Table 1:** The list of traditional Persian medical books from 9th century to 20th century AD used to investigate herbal medications for oligomenorrhea and amenorrhea.

Original title	Latin title	Author	Century produced (AD)	Subject
Al-Hawi fi-tibb [13]	Liber Continens	Abu Bakr Muhammad ibn Zakariyya al Razes (Rhazes)	9	A large medical encyclopedia on diseases, therapy, and pharmacology in twenty-five volumes

Al- Qanun fit-tib [11]	The Canon of Medicine	Ibn Sina (Avicenna)	11	An encyclopedia of medicine in five comprehensive volumes, a main medicine reference in the Western countries until the 16th century

Zakhireh Kharazmshahi [14]	Treasure of Khawarazm Shah	Seyyed Ismaeil Jorjani	11	A Persian medical encyclopedia in ten chapters

Kitāb al-Jāmi li-mufradāt al-adwiya wa-l-aghdhiya [15]	Compendium on Simple Medicaments and Foods	Diyā Al-Dīn Abū Muhammad Abdollāh Ibn Ahmad (Ibn al-Baitār,)	13	Traditional pharmaceutical encyclopedia containing the names and properties of more than 1,000 plants and substances of medicinal value

Al-asbāb wa-al-alāmāt [16]	Etiology and Symptoms [of Diseases]	Najīb al-Dīn Muhammad ibn 'Ali al-Samarqandī	13	Medical writing by emphasizing on causes and symptoms of diseases

Kitab at-Tasrifli-man 'ajaza 'an al-ta 'lif [17]	The Method of Medicine	Abu al-Qasim al Zahrawi (Abulcasis)	11	A 30-volume encyclopedia of medicine containing information about medical conditions, treatments, and surgical procedures

Tuhfat al-mu'minin [18]	The Present for the Faithful	Muhammad Mu'min Daylamī Tunakābunī	17	Major pharmaceutical books of ITM, a dictionary of plants, animals, and minerals

Makhzan ul-adviyyah [19]	The Storehouse of Medicaments	Muhammad Hāshim Hādī Alavī Shīrāzī	18	Major pharmaceutical books of ITM, a dictionary of plants, animals, and minerals

Exir azam [12]	Great Elixir	Hakim Mohammad Azam Khan	20	A medical textbook describes etiology, signs, and symptoms of diseases and their treatment

**Table 2:** Ranking the medicinal plants groups from the most efficient to the least efficient.

Number	Group	Description
(1)	A + C	Potent and frequent with evidence

(2)	A + D	Potent and frequent without evidence

(3)	B + C	Potent or frequent with evidence

(4)	B + D	Potent or frequent without evidence

(5)	E	Not potent or frequent Due to limitations and for higher efficiency, these plants were not searched in conventional medicine and excluded from the study, although studies may be found in this group.

**Table 3:** Summary of plants found in traditional Persian medicine and conventional medicine references.

Data	Total plants	Potent plants	Frequent plants	^*∗*^Potent and frequent plants (group A in flowchart of study)	^*∗∗*^Plants with evidences (group C in flowchart of study)	^*∗∗∗*^Potent and frequent plants with evidences (group 1 in ranking table)
NO	87	50	70	33	8	5

^*∗*^ *Allium ampeloprasum, Apium graveolens, Aristolochia fontanesii *Boiss. & Reut.*, Artemisia absinthium, Asarum europaeum, Brassica oleracea *L*., Cinnamomum cassia, Citrullus colocynthis, Commiphora mukul, Cyclamen purpurascens *Mill*., Daucus carota *L.*, Foeniculum vulgare *Mill*., Ferula gummosa, Ferula persica *Willd*., Helleborus niger, Hypericum perforatum *L*., Levisticum officinale, Lilium candidum, Mentha longifolia, Nigella sativa *L.*, Paeonia lactiflora Pall, Petroselinum crispum, Phaseolus vulgaris, Prangos ferulacea *L.*, Rubia tinctorum, Sesamum indicum *L.*, Tanacetum parthenium, Teucrium chamaedrys, Teucrium polium *L.*, Thymus vulgaris, Urtica dioica, Vitex agnus-castus *L., and* Ziziphora clinopodioides *Lam.;^*∗∗*^*Cinnamomum verum, Foeniculum vulgare, Mentha longifolia, Paeonia lactiflora, Sesamum indicum *L.*, Trigonella foenum-graecum, Urtica dioica, *and* Vitex agnus-castus*;^*∗∗∗*^*Foeniculum vulgare, Mentha longifolia, Paeonia lactiflora, Sesamum indicum *L*., *and* Vitex agnus-castus*.

**Table 4:** Medicinal plants used for treatment of oligomenorrhea and amenorrhea in traditional Persian medicine references.

Number	Traditional name	Suggested scientific name (synonym)^*∗*^	Family	^*∗∗*^Temperament	Part(s) used	Methods of application	^*∗∗∗*^Repetitive plants	^*∗∗∗∗*^Potent plants
(1)	Ghafes	*Agrimonia eupatoria* L. *Eupatorium cannabinum*	Rosaceae Asteraceae	Warm & dry	Aerial	Oral, vaginal (Hamool)	-	*∗*

(2)	Komaphytus	*Ajuga chamaepitys*	Lamiaceae	Warm & dry	Aerial	Oral, vaginal (Hamool)	5 P.B & 2 M.B	-

(3)	Koras	*Allium ampeloprasum* L. *(Allium porrum *L.) *Allium ascalonicum* L.. *Allium roseum* L. *Allium scorodoprasum* L. *Allium ursinum* L. *Allium vineale* L.	Amaryllidaceae Liliaceae Alliaceae	Warm & dry	Leaf, bulb	Oral	5 P.B & 3 M.B	*∗*

(4)	Basal	*Allium cepa *L.	Amaryllidaceae Liliaceae Alliaceae	Warm & dry	Bulb	Oral, bath	5 P.B & 2 M.B	-

(5)	Sooom	*Allium sativum *L.	Amaryllidaceae Liliaceae	Warm & dry	Bulb	Bath	5 P.B & 2 M.B	-

(6)	Abu khalsa	*Alkanna tinctoria* *(Anchusa tinctoria *L.)	Boraginaceae	Warm & dry	Root	Oral, vaginal (Hamool), bath	-	*∗*

(7)	Karafs	*Apium graveolens* *Petroselinum crispum *(Mill.)Fuss (*Apium petroselinum *L.)	Apiaceae	Warm & dry	Fruit, stem, root	Oral, vaginal (Hamool)	5 P.B & 3 M.B	*∗*

(8)	Zaravand	*Aristolochia fontanesii* Boiss. & Reut. (*Aristolochia longa* L.) Aristolochia bottae Jaub. & Spach. (*Aristolochia maurorum* L.)	Aristolochiaceae	Warm & dry	Aerial, root	Oral, vaginal (Forzajah)	5 P.B & 2 M.B	*∗*

(9)	Ghaysoom	*Artemisia abrotanum* *Artemisia montana *(Nakai)Pamp.	Asteraceae	Warm & dry	Flower, leaf, root	Oral, vaginal (Hamool)	5 P.B & 2 M.B	-

(10)	*Afsantin*	*Artemisia absinthium* *Artemisia maritima *L. *Artemisia sieversiana *Ehrh. ex Willd.	Asteraceae	Warm & dry	Aerial, leaf	Oral, vaginal (Forzajah)	5 P.B & 4 M.B	*∗*

(11)	Berenjasef	*Artemisia vulgaris *L. *Achillea eriophora *DC.	Asteraceae Compositae	Warm & dry	Root, aerial	Oral, bath, topical	5 P.B & 2 M.B	-

(12)	Asaron	*Asarum europaeum*	Aristolochiaceae	Warm & dry	Root, leaf	Oral	5 P.B & 3 M.B	*∗*

(13)	Helyoun	*Asparagus officinalis *L. *Asparagus adscendens *Roxb.	Asparagaceae	Warm & dry	Aerial, rhizome, root	Vaginal (Hamool & Forzajah)	5 P.B & 1 M.B	-

(14)	Komashir	*Athamanta macedonica *L.	Apiaceae	Warm & dry	Aerial	Oral, vaginal (Hamool)	5 P.B	-

(15)	Kornob	*Brassica oleracea *L.	Brassicaceae	Warm & dry	Leaf	Oral, vaginal (Hamool & Bakhoor)	5 P.B & 4 M.B	*∗*

(16)	Ghantoriyoun	*Centaurium erythraea*	Gentianaceae	Warm & dry	Aerial	Vaginal (Forzajah)	5 P.B & 2 M.B	-

(17)	Qost	*Cheilocostus speciosus (Costus speciosus)*	Costaceae	Warm & dry	Rhizome	Oral, vaginal (Forzajah & Bakhoor)	5 P.B & 2 M.B	-

(18)	Khandarili	*Chondrilla juncea *L.	Asteraceae	Dry	Leaf	Vaginal (Forzajah)	-	*∗*

(19)	Hemmes	*Cicer arietinum*	Fabaceae	Warm & dry	Seed	Oral	5 P.B & 3 M.B	-

(20)	Salikheh	*Cinnamomum cassia* *(Cinnamomum aromaticum)* *Cinnamomum iners* *Cinnamomum bejolghota *Sweet	Lauraceae	Warm & dry	Bark	Oral	5 P.B & 3 M.B	*∗*

(21)	Darcini	*Cinnamomum verum* (*Cinnamomum zeylanicum*)	Lauraceae	Warm & dry	Bark	Oral, vaginal (Hamool)	5 P.B & 4 M.B	-

(22)	Ladan	*Cistus ladanifer *L. *Cistus creticus *L. *Cistus incanus *L. *Tropaeolum majus *L.	Cistaceae Tropaeolaceae	Warm & dry	Flower, leaf, seed, whole plant	vaginal (Forzajah & Bakhoor)	5 P.B	-

(23)	Hanzal	*Citrullus colocynthis* (*Cucumis colocynthis *L.)	Cucurbitaceae	Warm & dry	Fruit	Vaginal (Forzajah & Bakhoor)	5 P.B & 3 M.B	*∗*

(24)	Otroj	*Citrus medica* L.	Rutaceae	Compound	Fruit	Oral	-	*∗*

(25)	Moghl	*Commiphora mukul *(Hook. ex Stocks) Engl.	Burseraceae	Warm & dry	Gum	Oral, vaginal (Bakhoor)	5 P.B & 1 M.B	*∗*

(26)	Morr	*Commiphora myrrha *(Nees) Engl. *(Commiphora molmol)*	Burseraceae	Warm & dry	Vaginal	Oral, vaginal (Hamool)	5 P.B & 1 M.B	-

(27)	Phaghlaminus/Artanisa	*Cyclamen purpurascens *Mill. *(Cyclamen europaeum)*	Primulaceae	Warm & dry	Rhizome, root	Oral, vaginal (Hamool)	5 P.B & 2 M.B	*∗*

(28)	Ezkher	*Cymbopogon citratus* *Cymbopogon jwarancusa *(Jones) Schult. *Cymbopogon nardus *(L.) Rendle	Poaceae	Warm & dry	Flower	Oral, topical	5 P.B & 3 M.B	-

(29)	Soed	*Cyperus longus* L. *Cyperus rotundus *L.	Cyperaceae	Warm & dry	Root	Oral	-	*∗*

(30)	Dooghou	*Daucus carota* L.	Apiaceae	Warm & dry	Root	Oral, vaginal (Hamool)	5 P.B & 2 M.B	*∗*

(31)	Oshagh	*Dorema ammoniacum*	Apiaceae	Warm & dry	Resin	Oral, vaginal (Hamool)	-	*∗*

(32)	Loof	Dracunculus vulgaris Schott (*Arum dracunculus *L.) *Arum italicum* *Arum maculatum*	Araceae	Warm & dry	Root	Oral, vaginal (Hamool), nasal	-	*∗*

(33)	Ghesa-al hemar	*Ecballium elaterium *L. *(Momordica elaterium)*	Cucurbitaceae	Warm & dry	Fruit, seed	Oral, vaginal (Forzajah)	5 P.B & 1 M.B	-

(34)	Kheiry	*Erysimum *×* cheiri *L. (*Cheiranthus cheiri *L.)	Brassicaceae	Warm & dry	flower	Oral, vaginal (Hamool), bath		*∗*

(35)	Farfiyoun	*Euphorbia helioscopia* L. *Euphorbia resinifera*	Euphorbiaceae	Warm & dry	Flowering plant, root	Oral, vaginal (Hamool)	-	*∗*

(36)	Razyanaj	*Foeniculum vulgare Mill*	Apiaceae	Warm & dry	Seed, root	Oral	5 P.B & 2 M.B	*∗*

(37)	Barzad	*Ferula gummosa*	Apiaceae	Warm & dry	Resin	vaginal (Hamool & Bakhoor)	5 P.B & 1 M.B	*∗*

(38)	Sakbinaj	*Ferula persica *Willd.	Apiaceae	Warm & dry	Resin	Oral, vaginal (Forzajah & Bakhoor)	5 P.B & 5 M.B	*∗*

(39)	Tin	*Ficus carica *L.	Moraceae	Warm & moist	Fruit	vaginal (Hamool)	5 P.B	-

(40)	Jentiana	*Gentiana lutea*	Gentianaceae	Warm & dry	Root	Oral	5 P.B & 2 M.B	-

(41)	Kondosh	*Gypsophila struthium Loefl* *Schoenocaulon officinale *(Schltdl. & Cham.) A.Gray *Veratrum album *L.	Caryophyllaceae Melanthiaceae	Warm & dry	Root	Oral, vaginal (Hamool)	-	*∗*

(42)	Ashagheh	*Hedera helix *L.	Araliaceae	Warm & dry	Leaf	Vaginal (Forzajah)	5 P.B	-

(43)	Kharbagh siyah	*Helleborus niger*	Ranunculaceae	Warm & dry	Rhizome, root	Vaginal (Forzajah)	5 P.B & 3 M.B	*∗*

(44)	Hofarighoon	*Hypericum perforatum L* *Hypericum barbatum Jacq* *Hypericum coris *L.	Hypericaceae	Warm & dry	Bud, flower, aerial	Oral, vaginal (Hamool)	5 P.B & 1 M.B	*∗*

(45)	Rasan	*Inula helenium* *Calamintha incana* Boiss.	Asteraceae Lamiaceae	Warm & dry	Rhizome	Oral, vaginal (Bakhoor)	5 P.B & 4 M.B	-

(46)	Irsa	*Iris Germanica* *(Iris × florentina L.)* *Iris ensata *Thunb.	Iridaceae	Warm & dry	Rhizome, root	Oral, vaginal (Forzajah)	5 P.B & 1 M.B	-

(47)	Abhal	*Juniperus sabina *L.	Cupressaceae	Warm & dry	berry cones, pseudo fruit	Oral, vaginal (Hamool & Bakhoor)	-	*∗*

(48)	Horf	*Lepidium sativum*	Brassicaceae	Warm & dry	Aerial	Oral	5 P.B & 3 M.B	-

(49)	Kashem/Anjedan roomi	*Levisticum officinale* *(Ligusticum levisticum)* *Seseli tortuosum*	Apiaceae	Warm & dry	Fruit, root, aerial	Oral	5 P.B & 3 M.B	*∗*

(50)	Soosan sefid	*Lilium candidium*	Liliaceae	Hot & mild	Bulb	Oral, vaginal (Hamool)	5 P.B & 1 M.B	*∗*

(51)	Maye-sayeleh	*Liquidambar orientalis *Mill.	Altingiaceae	Warm & dry	Sap	Oral, vaginal (Hamool & Forzajah & Bakhoor)	5 P.B & 1 M.B	-

(52)	Farasiyoun	*Marrubium vulgare*	Lamiaceae	Warm & dry	Aerial, whole herb	Oral, bath, topicl	5 P.B & 1 M.B	-

(53)	Baboonaj	*Matricaria chamomilla L.* *(Matricaria recutita)* *Anthemis nobilis *L. *Tripleurospermum disciforme *(C.A.Mey.)Sch.Bip.	Asteraceae	Warm & dry	flower	Oral, bath	5 P.B & 4 M.B	-

(54)	Sisanbar	*Mentha aquatica *L. *Mentha piperita *L.	Lamiaceae	Warm & dry	Aerial	Oral, bath	5 P.B & 2 M.B	-

(55)	Foodenj	*Mentha pulegium *L. *Mentha longifola *L. *Mentha aquatica *L. *Mentha × piperita *L.	Lamiaceae	Warm & dry	Aerial, leaf	Oral, vaginal (Hamool)	5 P.B & 5 M.B	*∗*

(56)	Shoniz	*Nigella sativa *L.	Ranunculaceae	Warm & dry	Seed	Oral	5 P.B & 3 M.B	*∗*

(57)	Jawshir	*Opopanax chironium* *Prangos ferulacea* (L.) Lindl.	Apiaceae	Warm & dry	Gum	Oral, vaginal (Hamool)	5 P.B & 5 M.B	*∗*

(58)	Fawania	*Paeonia lactiflora Pall*	Paeoniaceae	Warm	Seed, root	Oral	5 P.B & 2 M.B	*∗*

(59)	Harmal	*Penagum harmala* *(Harmala peganum)*	Nitrariaceae	Warm & dry	Seed	Oral, topical		*∗*

(60)	Fatrasaliyoun	*Petroselinum crispum* *(Carum Petroselinum, Petroselinum sativum)*	Apiaceae	Warm & dry	Fruit, aerial	Oral	5 P.B & 3 M.B	*∗*

(61)	Loobia	*Phaseolus vulgaris*	Leguminosae	Warm & moist	Seed	Oral, bath	5 P.B & 4 M.B	*∗*

(62)	Anison	*Pimpinella anisum*	Umbelliferae	Warm & dry	Fruit	Oral, vaginal (Hamool & Bakhoor)	5 P.B & 2 M.B	-

(63)	Senobar	*Pinus* sp.	Pinaceae	Warm & dry	Resin	Vaginal (Bakhoor)	-	*∗*

(64)	Felfel	*Piper nigrum *L.	Piperaceae	Warm & dry	Fruit	Oral, vaginal (Hamool)	5 P.B & 2 M.B	-

(65)	Gharasia	*Prunus cerasus *L. *(Cerasus vulgaris)* *Prunus avium *L. *(Cerasus avium)*	Rosaceae	Cold & dry	Fruit, peduncle	Oral	-	*∗*

(66)	Loos al mor	*Prunus dulcis* *(Amygdalus communis var. amara)*	Rosaceae	Warm & dry	Leaf, flower, fruit	Vaginal (Hamool & Forzajah)	5 P.B & 3 M.B	-

(67)	Fowweh	*Rubia tinctorum*	Rubiaceae	Warm & dry	Root	Vaginal (Hamool)	5 P.B & 3 M.B	*∗*

(68)	Hommaz	*Rumex acetosa *L. *Rumex conglomeratus Murray* (*Rumex acutus *Sm.)	Polygonaceae	Cold & dry	Leaf, aerial	Oral	-	*∗*

(69)	Sodab	*Ruta graveolens* L.	Rutaceae	Warm & dry	Aerial	Vaginal (Hamool & Forzajah)		*∗*

(70)	Satroniyoun	*Saponaria officinalis*	Caryophyllaceae	Warm & dry	Root	Oral, vaginal (Hamool)	-	*∗*

(71)	Oshnan	*Seidlitzia rosmarinus* *Salicornia *sp.	Amaranthaceae	Warm & dry	Leaf, stem, ash	Oral	5 P.B & 3 M.B	-

(72)	Semsem	*Sesamum indicum *L. *(Sesamum orientale *L.)	Pedaliaceae	Warm & moist	Seed	Oral	5 P.B & 1 M.B	*∗*

(73)	Ghora-tol-ayn	*Sium latifolium L*	Apiaceae	Warm & dry	Root	Oral	5 P.B	-

(74)	Astarak	*Styrax officinale*	Styracaceae	Warm & dry	Resin	Oral, vaginal (Forzajah & Bakhoor)	5 P.B & 1 M.B	-

(75)	Ogh'hovan	Tanacetum parthenium *Anthemis arvensis *L. *Anthemis cotula *L.	Compositae	Warm & dry	Aerial	Oral, vaginal (Forzajah)	5 P.B	*∗*

(76)	Khas barri	*Taraxacum campylodes *G.E.Haglund *(Taraxacum officinale)*	Asteraceae	Cold & moist	Aerial, leaf, rhizome, root, whole herb	Oral	5 P.B	-

(77)	Kamadarius	*Teucrium chamaedrys*	Lamiaceae	Warm & dry	Aerial	Oral	5 P.B & 2 M.B	*∗*

(78)	Jo'dah	*Teucrium polium *L.	Lamiaceae	Warm & dry	Whole herb	Oral	5 P.B	*∗*

(79)	Hasha	*Thymus vulgaris* *Thymus capitatus*	Lamiaceae	Warm & dry	Aerial, leaf	Oral	5 P.B & 4 M.B	*∗*

(80)	Holbeh	*Trigonella foenum-graecum*	Fabaceae	Warm & dry	Seed	Oral, bath, topical	5 P.B & 3 M.B	-

(81)	Handaghughi	*Trifolium pretense* *Trigonella coerulea *(Desr.) Ser.	Leguminosae	Warm & dry	Flower	Oral	5 P.B & 2 M.B	-

(82)	Anjoreh	*Urtica dioica*	Urticaceae	Warm & dry	Flowering plant, root	Oral, vaginal (Hamool & Fetelah)	5 P.B	*∗*

(83)	Phow	*Valeriana dioscoridis *Sm.	Caprifoliaceae	Warm & dry	Aerial, rhizome	Oral, bath	5 P.B	-

(84)	Kharbagh sefid	*Veratrum album L.*	Melanthiaceae	Warm & dry	Root, rhizome	Vaginal (Forzajah)	5 P.B & 3 M.B	-

(85)	Aslagh	*Vitex agnus-castus* L.	Lamiaceae	Warm & dry	Fruit, leaf	Oral, vaginal (Hamool & Bakhoor)	5 P.B & 2 M.B	*∗*

(86)	Mo	*Vitis vinifera *L. *(Vitis sylvestris *C.C.Gmel)	Vitaceae	Warm & dry	Leaf	Oral, bath	5 P.B	-

(87)	Meshketaramashie	*Ziziphora clinopodioides *Lam. *Origanum dictamnus* *Mentha aquatic *L.	Lamiaceae	Warm & dry	Leaf	Oral, vaginal (Bakhoor)	5 P.B & 4 M.B	*∗*

^*∗*^References were regarding scientific names [20–25]. ^*∗∗*^According to TPM basics, four provital elements as fire, air, water, and soil are the main components of all objects in the world. Every element has particular quality: fire is hot and dry, air is hot and wet, water is cold and wet, and soil is cold and dry. Difference and variety of objects are based on the different amount of these elements in their structures. After action and reaction of four main elements, one or two qualities will be overcoming. Temperament is actually specific quality of an object based on dominant element(s) in its structure and creates different characteristics in objects. All things in the world, including humans, plants, and even diseases have temperaments [10, 26]. ^*∗∗∗*^Repetitious plants: repeated at least in 5 pharmaceutical textbooks of traditional Persian medicine: Al-Hawi al- kabir, Al- Qanun fit-tib, Al- Jame lemofradat al- aghziyeh va al- adviyeh, Tuhfat al-mu'minin, and Makhzanal al- adviyeh. ^*∗∗∗∗*^Experienced, specific, unique plants: medicinal plants which were special or potent or experienced in menstrual induction according to traditional Persian medicine scientists' concept (mentioned in Al-Havi al- kabir, Al- Qanun fit-tib, Al- Jame lemofradat al- aghziyeh va al- adviyeh, Tuhfat al-mu'minin, Makhzanal al- adviyeh, and Al-Tasrif). Hamool or vaginal or rectal cotton-load: a tissue of cotton or wool loading with natural drugs was inserted in the vagina or rectum [27]. Forzajah or vaginal pessary: a wet cotton or wool tissue feeding with dry or wet compound natural medications placed inside vagina [27]. Fateelah or vaginal or rectal wick: a strip or thread of fibrous or spongy material feeding with special medications inserted into the penis fossa, rectum, or vagina [27]. Bakhoor or vaginal or rectal fumigation: the smoke of burning natural drugs [27]; P.B: pharmaceutical textbooks of traditional Persian medicine (as Al-Hawi al- kabir, Al- Qanun fit-tib, Al- Jame lemofradat al- aghziyeh va al- adviyeh, Tuhfat al-mu'minin, and Makhzanal al- adviyeh); M.B: medical textbooks of traditional Persian medicine (Al-Hawi al- kabir, Al- Qanun fit-tib, Al-Tasrif, Zakhireh Kharazmshahi, Al-asba*¯*b wa-al-ala*¯*ma*¯*t, and Exir Azam).

**Table 5:** Studies on medicinal plants used for treatment of oligomenorrhea and amenorrhea.

Number	Authors/country/year	Scientific name	Part/compound	Design	Participant	Sample size	Intervention protocol	Duration of study	Results
(1)	Kort and Lobo Columbia 2014 [28]	*Cinnamomum verum *	Supplements (Cinnulin PF; Integrity Nutraceuticals International, Spring Hill, TN)	Prospective, placebo controlled, double-blinded randomized trial	PCOs patients 18–38 years in two groups; treatment (*n* = 23) and placebo (*n* = 22)	45	1.5 g/d supplements or placebo (4 capsules of 125 mg, TDS)	6 months	Significant improvement in menstrual cyclicity in cinnamon group compared with baseline and placebo. Menstrual bleeding was resulted from ovulatory cycle because of luteal phase progesterone levels (>3 ng/ml) in 5 samples in the cinnamon group No considerable changes in markers of insulin resistance, serum androgen, SHBG levels, weight and ovarian volume in both groups.

(2)	Mohebbi-Kian et al. Iran 2014 [29]	*Foeniculum vulgare *Mill.	Essential oil from fennel seeds (containing 71–90 mg anethole)	Double-blind double-dummy randomized, placebo-controlled, parallel trial	15–45 years married women using DMPA and without menstrual bleeding for 45–140 days prior in three groups; fennel essential oil (FEO, *n* = 26), LD-COC (*n* = 26) and placebo (*n* = 26)	78	Each pack contained 21 LD-COC pills + 42 placebo capsules, 21 placebo pills + 42 fennel capsules, 21 placebo pills and 42 placebo capsules One pill and a capsule BID	21 days	Experience of menstrual bleeding in 73% of women in the FEO and 81% of women in LD-COC groups which markedly higher than the placebo group (19%), but no significant alteration between fennel and LD-COC groups ↑ mean amount of menstrual bleeding in the FEO group (21 cc) compared to the LD-COC (14 cc) and placebo (12 cc) groups ↑ duration of menstrual bleeding/spotting and the number of used sanitary pads in the FEO group (5.2 days, 10.4 pads) compared to the LD-COC (4 days, 7.4 pads) and placebo (4 days, 6.8 pads) groups ↓ mean duration of drug using in fennel group (14 days) compared to LD-COC (21 days) and placebo (20 days) groups (*p* < 0.001) ↑ injections of DMPA after intervention in fennel (73%) and LD-COC groups (65%) compared to placebo group (31%).

(3)	Mokaberinejad et al. Iran 2012 [30]	*Mentha longifolia*	Ethanolic extract of dried plant powder prepared as syrup	Double-blind, randomized, placebo-controlled, multi center study	18–35 years women with secondary amenorrhea and oligomenorrhea (*N* = 120) in two groups; treatment (*n* = 60) and placebo (*n* = 60)	120	45 ml of plant extract or placebo syrup TDS (in treatment group: 300 mg ethanol extract of plant in 5 ml of syrup)	three menstrual cycles	68.3% patients in the treatment and 13.6% in the placebo group experienced uterus bleeding during the first cycle (*p* < 0.001), regular bleeding during three cycles in one third of the patients treated with the herbal syrup (33.3%) compared to placebo (3.3%). ↓ LH in treatment group compared to placebo (*p* < 0.002), no changes in other hormonal parameters in both groups.

(4)	Mokaberinejad et al. Iran 2014 [31]	*Mentha longifolia*	Herbal tea of dried leaves	Pilot study, before & after	Amenorrheic 30–40 years old women with POF	27	A 250 mL cup of herbal tea (250 mL of boiling water over 2 g of the dried leaves) TDS	2 weeks	↓ FSH (*p* < 0.001), occurrence of menstrual bleeding in all patients except four on average 19.2 days after taking the medication (*p* < 0.001).

(5)	Ushiroyama et al. Osaka 2001 [32]	*Paeonia lactiflora* with *Cinnamomum cassia*	Unkei-to	Randomized controlled clinical trial	Anovulatory women with high plasma LH levels (PCOs = 38 and non PCOs = 62) in two groups; control (*n* = 48) and treatment (*n* = 52)	100	Not mentioned	8 weeks	↓ LH (mean rate = 22.2 ± 35.7% in PCOs and 49.7 ± 15.3% in non- PCOs patients) and ↑ estradiol in unkei-to treatment group Development of the dominant follicle in patients treated with unkei-to. Improvement in menstrual cyclicity (50% in PCOs and 60% in the non-PCOs group) in unkei-to treatment group but no significant difference between the two groups.

(6)	Yavari et al. Iran 2014 [33]	*Sesamum indicum *L.	Powder	Pilot study	20 to 40 years old women with oligomenorrhea and complaint of more than 2 weeks menstruation retard	21	60 g powdered with a tea spoon of honey once daily before breakfast	7 days	Experience of menstrual bleeding in 85% of the patient after treatment within two weeks Higher volume of menstrual bleeding in 20% (*n* = 4) of the patient after treatment Drug-free episode of menstruation in 80% of the patient in less than 2 weeks.

(7)	Yavari et al. Iran 2016 [34]	*Sesamum indicum *L.	Powder	Single blind randomized controlled clinical trial	Women with oligomenorrhea in two groups; progesterone (*n* = 29) and sesame group (*n* = 27)	56	60 g sesame powdered once daily or Medroxy Progesterone 5 mg tablets BID	A week	Occurrence of menstrual bleeding in 72% of the patient in the sesame group and 93.10% in the progesterone group (significantly higher than the sesame group, *p* = 0.012) No marked increasing in volume of blood flow and severity of pain in both groups ↓ duration of drug using for experiencing menstrual bleeding in sesame group compared to progesterone group (10.38 days versus 11.8) On-time menstruation in drug-free episode in 50% of the patients in sesame group compared to 6% in progesterone group.

(8)	Bashtian et al. Iran 2013 [35]	*Trigonella foenum-graecum*	Hydroalcoholic extract of seeds	Prospective randomized, double-blind, placebo-controlled trial	20–35 year-old women with PCOs + menstrual disturbances and infertility ± clinical signs of hyperandrogenism chief complaints in two groups; treatment (*n* = 30) and placebo (*n* = 28)	58	500 mg of extract or placebo BID plus metformin TDS	8 weeks	↓ significant in polycystic-appearing ovaries in ultrasound scans in extract group after treatment (*p* = 0.01) No significant changes in BMI, markers of insulin resistance (HOMA-IR) and insulin sensitivity (QUICKI), testosterone and 17-*α* OHP levels, F-G score in both groups. Normalizing menstrual cycle in 12 women with oligo-amenorrhea in extract group.

(9)	Swaroop et al. India 2015 [36]	*Trigonella foenum-graecum*	A patent-pending water-ethanol extract of seeds	Open-label, single arm, non-randomized, clinical study	Premenopausal women (18–45 years) with PCOs	50	2 capsule of 500 mg daily	3 months	↑ LH (*p* = 0.045) and ↑ FSH (*p* = 0.010), ↓ LH/FSH ratio (3.16 to 1.61), but not significant ↓ left & right ovary volume (↓17.82% & 28.25%), ↓ cyst size in 47 subjects, no cyst in 36 subjects, regular cycles in 71% of subjects, ↑ Hb levels, ↓ ALP, no significant change in WBC, AST, ALT, BUN and creatinine and 12% pregnancy after treatment compared to the baseline.

(10)	Najafipour et al. Iran 2014 [37]	*Urtica dioica*	Dried extract of root	Randomized controlled clinical trial	Hyperandrogenism female in two groups; experimental (extract, *n* = 20) and control (standard treatment, *n* = 20)	40	300–600 mg of plant dried extract or cyproterone compound and Spironolactone	4 months	↓ total, free testosterone (*p* = 0.002) and DHEA (*p* = 0.063) after treatment in the experimental group No significant difference between the study parameters (total, free testosterone and DHEAS) in two groups ↑ improvement of acne, greasy skin (*p* < 0.001), menstrual cycle situation (*p* = 0.044) in the control group compared to the experimental group after the treatment.

(11)	Bergmann et al. Germany 2000 [38]	*Vitex agnus-castus *	Homeopathic preparation (*Silybum marianum*, *Vitex agnus-castus*, *Chelidonium majus*)	Randomized, placebo-controlled clinical double-blind study	Women with oligomenorrhea (*n* = 37) or amenorrhea (*n* = 30) in two groups; treatment and placebo	67	50 drops of Phyto Hypophyson L or placebo TDS	3 months or 3 cycles	Occurrence of menstruation and shortening of the cycle in the treatment group compared to the placebo. ↑ luteal phase progesterone in oligomenorrheal women after treatment relative to placebo. Improvement of ovulation and 38 pregnancy out of 67 women in the treatment group compared to the placebo.

(12)	Shahnazi et al. Iran 2016 [39]	*Vitex agnus-castus*	Fruit extract	Randomized, triple-blind clinical trial with a placebo controlled	Women 18–45 years old with PCOS and oligomenorrhea or amenorrhea in two equal groups; LD and *Vitex agnus-castus* groups	70	Capsule contained LD pill or extract daily	3 months	Normalization the menstrual cycle duration in 68.6% of the LD group members and 60% of the extract participants without considerable difference between the two groups (*p* = 0.45) ↓ means of the free testosterone, prolactin and DHEAS level in the LD and the extract groups after treatment, but no significant difference between the two groups.

↑: increase, ↓: decrease, PCOs: polycystic ovary syndrome, BMI: body mass index, FSH: follicle-stimulating hormone, LH: luteinizing hormone, DHEAS: dehydroepiandrosterone sulfate, SHBG: sex hormone-binding globulin, HOMA-IR: homeostasis model assessment for insulin resistance, QUICKI: quantitative insulin sensitivity check index, WBC: white blood cells, Hb: hemoglobin, AST: aspartate aminotransferase, ALT: alanine aminotransferase, ALP: alkaline phosphatase, BUN: blood urea nitrogen, F-G: Ferriman–Gallwey score, LD-COC: low dose combined oral contraceptive, and DMPA: depot medroxyprogesterone acetate.
